# Basal forebrain somatostatin cells differentially regulate local gamma oscillations and functionally segregate motor and cognitive circuits

**DOI:** 10.1038/s41598-019-39203-4

**Published:** 2019-02-22

**Authors:** Nelson Espinosa, Alejandra Alonso, Ariel Lara-Vasquez, Pablo Fuentealba

**Affiliations:** Departamento de Psiquiatria, Centro Interdisciplinario de Neurociencia, Pontificia Universidad Catolica de Chile, Santiago, Chile

## Abstract

The basal forebrain delivers extensive axonal projections to the cortical mantle regulating brain states and cognitive processing. Recent evidence has established the basal forebrain as a subcortical node of the default mode network that directionally influences cortical dynamics trough gamma oscillations, yet their synaptic origin has not been established. Here, we used optogenetic stimulation and *in vivo* recordings of transgenic mice to show that somatostatin neurons exert an anatomically specialized role in the coordination of subcortical gamma oscillations of the rostral basal forebrain. Indeed, the spike timing of somatostatin cells was tightly correlated with gamma oscillations in the ventral pallidum, but not in the medial septum. Consequently, optogenetic inactivation of somatostatin neurons selectively disrupted the amplitude and coupling of gamma oscillations only in the ventral pallidum. Moreover, photosupression of somatostatin cells produced specific behavioral interferences, with the ventral pallidum regulating locomotor speed and the medial septum modulating spatial working memory. Altogether, these data suggest that basal forebrain somatostatin cells can selectively synchronize local neuronal networks in the gamma band directly impinging on cortical dynamics and behavioral performance. This further supports the role of the basal forebrain as a subcortical switch commanding transitions between internally and externally oriented brain states.

## Introduction

The mammalian basal forebrain is a collection of subcortical structures which provides extensive axonal projections to the entire cerebral cortex^1,2^ playing central roles in regulating cognition, movement, brain states^3–8^ and consequently damage to the basal forebrain is critical in major neurological disorders^9–12^. The cortical action of the basal forebrain relies on the complementary roles of a heterogeneous mixture of three main cell populations: cholinergic, GABAergic, and glutamatergic cells^13^. Importantly, GABAergic cells are divided into at least two different cell types, pavalbumin-expressing and somatostatin-expressing cells^2,13–17^. Parvalbumin cells have been intensely studied in several brain regions including the cortex, thalamus, hippocampus, and basal forebrain^18–23^. Until recently, there was little information available on the circuit roles of somatostatin cells. Nonetheless, it is now known that they powerfully inhibit all other cell types in the basal forebrain and are able to gate basal forebrain synaptic output to the cortex^5,17^.

Recent evidence has demonstrated that basal forebrain gamma oscillations are enhanced during quiet wakefulness and self-grooming, which are internally-oriented states characteristic of the default mode network^24^. This stands in contrast with the canonical role of the basal forebrain in promoting active sensory processing and goal-directed behavior. Importantly, the prominent basal forebrain gamma oscillations are functionally and directionally coupled with cortical gamma-band activity, particularly in the prefrontal cortex^24^, which is a node of the default mode network^25^. Nevertheless, the circuit basis of such subcortical gamma oscillations has not yet been revealed. We have recently shown how somatostatin cells can gate basal forebrain synaptic output and regulate prefrontal cortex dynamics, with specific effects on gamma oscillations^26^. This posits somatostatin cells as a plausible candidate for the coordination of basal forebrain gamma oscillations. Accordingly, in the present study we set out to identify the role of somatostatin cells in the promotion of local gamma-band activity in two main domains of the rostral basal forebrain, the ventral pallidum and medial septum. Interestingly, we found anatomically segregated actions of somatostatin neurons, with only pallidal cells synchronizing subcortical gamma oscillations. Nevertheless, somatostatin cells in both regions exerted complementary roles on the regulation of exploratory behavioral patterns. Overall, our study further confirms the role of the basal forebrain as a dynamic switch between internally and externally oriented brain states.

## Results

### Spike timing of somatostatin cells correlates with rostral basal forebrain gamma band activity

**Figure 1:**
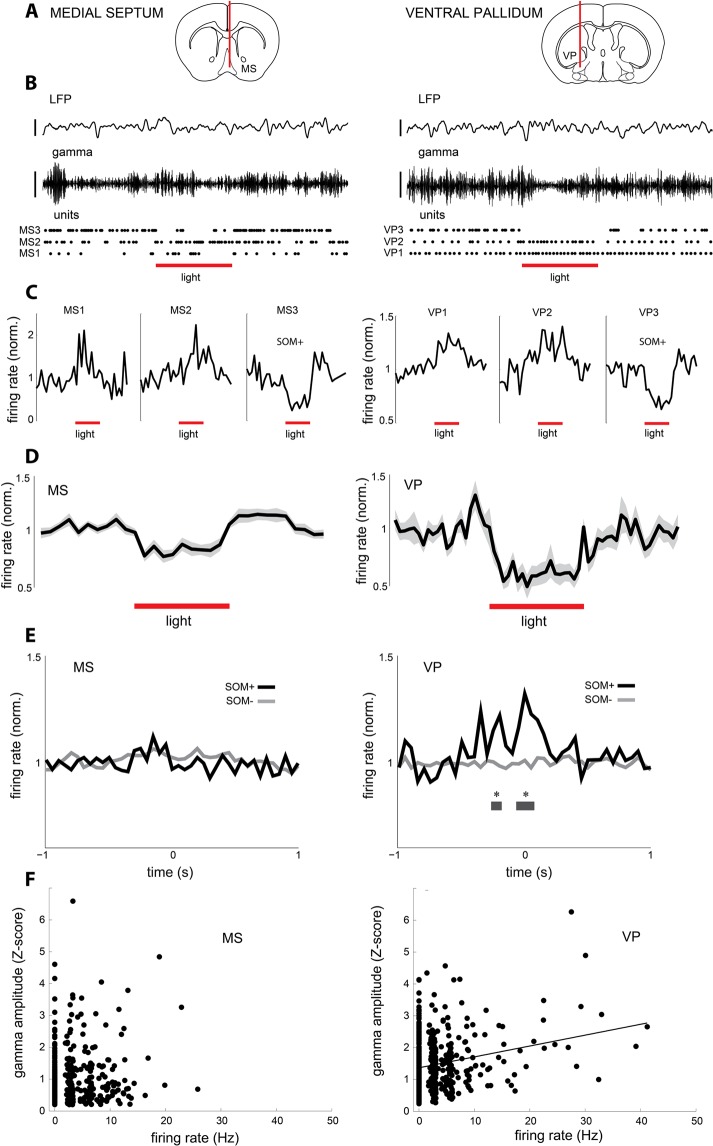
Spike timing of somatostatin cells is associated with rostral basal forebrain gamma oscillations. (**A**) Recording locations from different experiments represented in schematic coronal brain sections. Red lines represent the position of optrodes stereotaxically implanted in the medial septum (MS) or ventral pallidum (VP). (**B**) Multielectrode recordings of basal forebrain activity in anesthetized mice. Top, example channel of wideband LFP; middle, gamma oscillations filtered from the previous LFP channel (LFP 20–40 Hz); bottom, example single units responsive to laser stimulation. Every dot represents an action potential discharged by the cell. Note decreased gamma band activity during photostimulation in the VP. Laser pulse, 480 nm, 15 mW. Scale; LFP, 0.5 mV; gamma, 0.1 mV; light, 5 s. (**C**) Peri-event time histograms for the units shown in B (MS, 32 trials; VP, 17 trials). Cells 1 and 2 are excited (somatostatin-negative, SOM−), while cells 3 are inhibited (somatostatin-positive, SOM+) in both basal forebrain domains. (**D**) Average normalized discharge probability for all SOM + cells recorded in each basal forebrain domain (MS, n = 13; VP, n = 29) during optogenetic stimulation. Shading depicts standard error. (**E**) Crosscorrelograms of spiking activity from all recorded single units in relation to local gamma events, sorted as SOM+ (MS, n = 13; VP, n = 29) or SOM- (MS, n = 164; VP, n = 301). Note different discharge probability during gamma oscillations between SOM+ and SOM− cells in VP. Gray bar, Wilcoxon signed rank, P < 0.05. FDR, P = 0.0029. Furthermore, VP SOM+ cells’ correlograms were significantly different from chance (79% of cells, 500 shufflings). (**F**) Scatter plots and regression line for the firing rate of basal forebrain cells during gamma events versus the magnitude of gamma oscillations during which neurons were active (basal firing rate > 0.8 Hz, interquartile range). A significant Pearson correlation was detected only in the VP (r = 0.23, P = 1.7 × 10^−7^, n = 502 gamma events, 4 mice. MS, r = 0.095, P = 0.055, n = 409 gamma events, 7 mice).

We stereotaxically implanted an optrode into either the VP or MS of anesthetized transgenic animals (Fig. 1A) selectively expressing functional NpHR in somatostatin cells (+NpHR). We used a transgenic animal model to selectively inactivate somatostatin neurons^26^. Somatostatin cells were identified *in vivo* by conspicuous inhibition of their spiking activity during photostimulation in two domains of the rostral basal forebrain: the ventral pallidum (VP) and medial septum (MS) (Fig. 1C, Supplementary Table 1). As previously described^26^, only a minor fraction of recorded cells in the VP were somatostatin neurons (8.8%, n = 29), exhibiting significant suppression upon photostimulation (49.2 ± 5.1%). The remaining vast majority of neurons either increased its activity (17%, n = 56), presumably by synaptic disinhibition, or was not affected by laser stimulation (74.2%, n = 245). Similarly, in the MS, a small fraction of neurons was inhibited by photostimulation (7.3%, n = 13). The largest proportion of septal neurons either increased its activity (21.5%, n = 38) or was not affected by photostimulation (71.2%, n = 126). Excited and non-responsive cells belong to several different cell types, yet we operationally defined them as somatostatin-negative cells in order to simplify analysis (Fig. 1C). Thus, somatostatin cells could be functionally identified in two different domains of the rostral basal forebrain (Fig. 1D).

Prominent gamma oscillations of local synaptic origin have been described in the basal forebrain during different states of vigilance, which are particularly relevant to organize cortical dynamics in the default mode network^24^. In fact, filtering in the appropriate frequency band we could detect robust gamma oscillations in the rostral basal forebrain (Fig. 1A). Gamma oscillations in the rostral basal forebrain were not homogeneous. Indeed, they showed similar frequency range but differed in amplitude between the MS and VP (Supplementary Fig. 1, Supplementary Table 2). Next, we tested whether the activity of basal forebrain somatostatin cells was related to local gamma band oscillations. We analytically detected episodes of gamma band activity in the LFP^27–29^ and correlated them with the spike timing of basal forebrain neurons. We found that somatostatin cells discharged in close temporal relation with local gamma oscillations in the basal forebrain. However, only pallidal somatostatin cells were synchronized with gamma episodes, while septal somatostatin cells did not exhibit temporal correlation with the rhythm (Fig. 1E). In addition, the spike timing of somatostatin-negative cells was not correlated to gamma band activity in either basal forebrain domain (Fig. 1E). Finally, the firing discharge of pallidal neurons was positively correlated with the amplitude of gamma episodes (r = 0.23, P = 1.7 × 10^−7^), which was not the case for septal cells (P = 0.055, Fig. 1F). Taken together, these results suggest that ventral pallidum somatostatin cells discharge in close temporal relation with gamma oscillations in the rostral basal forebrain.

### Optogenetic inactivation of somatostatin cells disrupts rostral basal forebrain gamma oscillations

**Figure 2:**
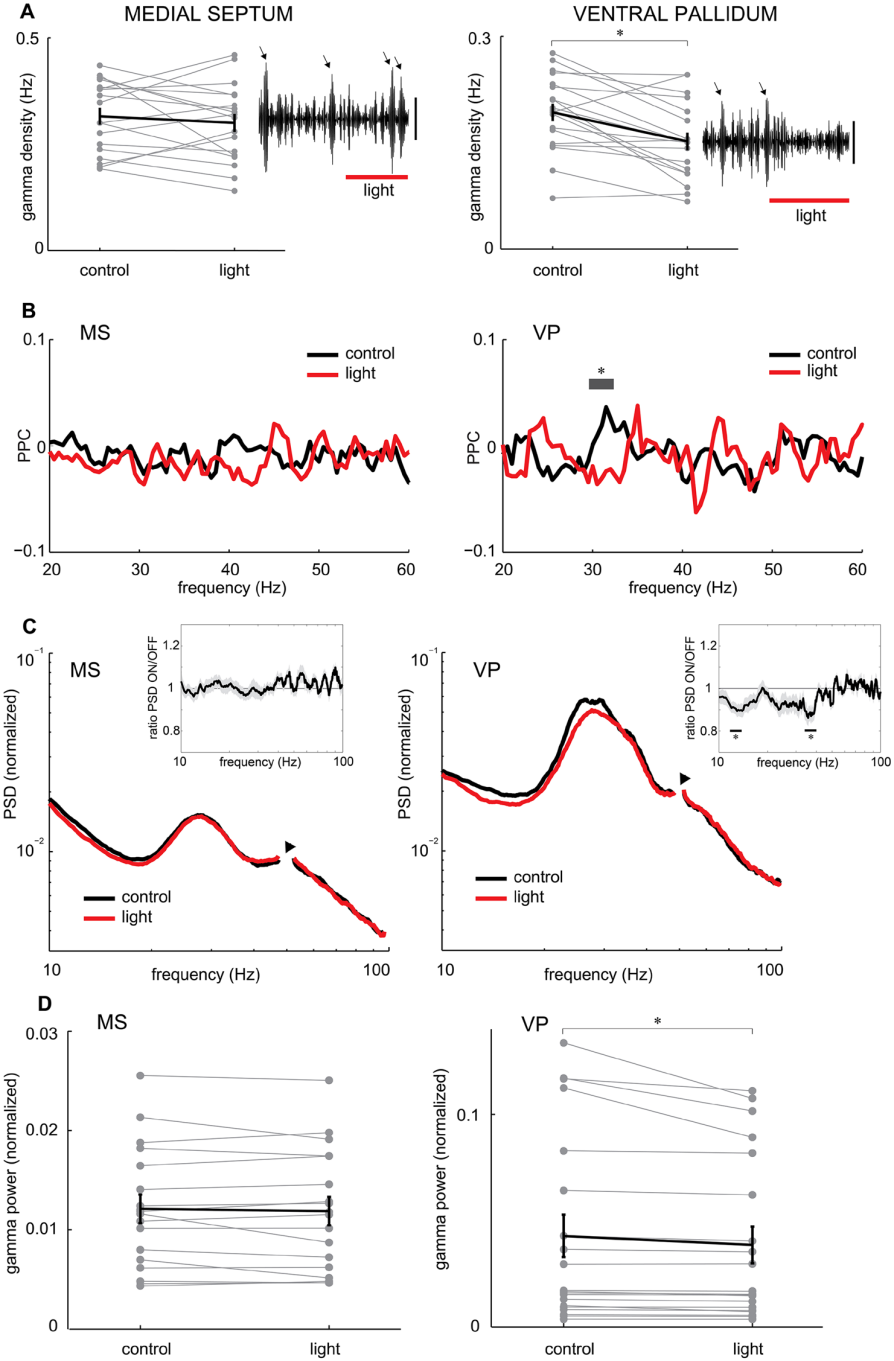
Photosupression of somatostatin cells impairs rostral basal forebrain gamma oscillations. (**A**) Density of gamma events during baseline (control) and during photostimulation (light) in the basal forebrain (MS, P = 0.39; VP, P = 2.3 × 10^−3^; paired t-test). Black lines, averages; gray lines, individual cases; asterisks, P < 0.05. Insets, examples of gamma band activity (LFP filtered 20–40 Hz) and gamma events (arrows), and the effect of photostimulation upon them (light). Note decreased power in the VP. Scale, 50 µV. (**B**) Average pairwise phase consistency (PPC) spectrum between SOM+ cells and LFP in control conditions (black line) and photostimulation (gray line). Note significant decrease in the gamma band (30 Hz) during photostimulation in VP (Wilcoxon signed-rank test, asterisk, P < 0.05). FDR, P = 0.0063. (**C**) Average normalized power spectral density (PSD) of the LFP recorded in the basal forebrain. Note narrow frequency range to highlight the gamma band. Inset, mean ratio between spectral distributions (PSD-laser on/PSD-laser off) for VP and MS. Gray bars depict significant differences (two-sided Wilcoxon signed-rank test; asterisk, P < 0.05). FDR, P = 0.0068. Shading depicts standard error. Arrowheads illustrate removal of 50 Hz artifact from the spectral distribution. (**D**) Plot of the effect of optogenetic stimulation on spontaneous gamma power on the basal forebrain (MS, n = 18, P = 0.37; VP, n = 20, P = 0.024; paired t-test; asterisk, P < 0.05).

Given their tight temporal relation, we next sought to establish the contribution of somatostatin cells to the generation of basal forebrain gamma oscillations. To this end, we quantified the effect of photostimulation on the density of oscillatory gamma events and found a significant decrease upon inactivation of somatostatin cells selectively in the VP (t-test, P = 2.3 × 10^−4^), but not in the MS (Fig. 2A). Given the close temporal relation between gamma oscillations and spiking timing of somatostatin cells, we tested their phase coupling. To this end, we used the pairwise phase consistency as a metric of oscillatory phase-locking between single neuron discharge and LFP activity^30^. Importantly, the phase-locking of pallidal somatostatin cells to gamma oscillations was significantly decreased by photostimulation (Fig. 2B). Conversely, septal somatostatin cells and somatostatin-negative neurons were poorly coupled to spontaneous gamma oscillations, a condition that was not affected by laser stimulation (Fig. 2B). Hence, the partial suppression of somatostatin cells by photostimulation altered their coupling to gamma oscillations in the VP, but not in the MS. We also quantified power in the different frequency bands of the basal forebrain LFP. The LFP frequency spectrum showed a prominent shoulder in the gamma range (20–40 Hz), which amplitude selectively decreased during photostimulation in the VP, but not in the MS (Wilcoxon signed rank test, P = 0.024, Fig. 2C,D). Overall, these results suggest that somatostatin cells in the rostral basal forebrain exert anatomically specific effects on local gamma band activity, with pallidal somatostatin neurons regulating both the amplitude and phase-locking of gamma cycles.

### Rostral basal forebrain somatostatin cells differentially regulate motor and cognitive circuits

**Figure 3:**
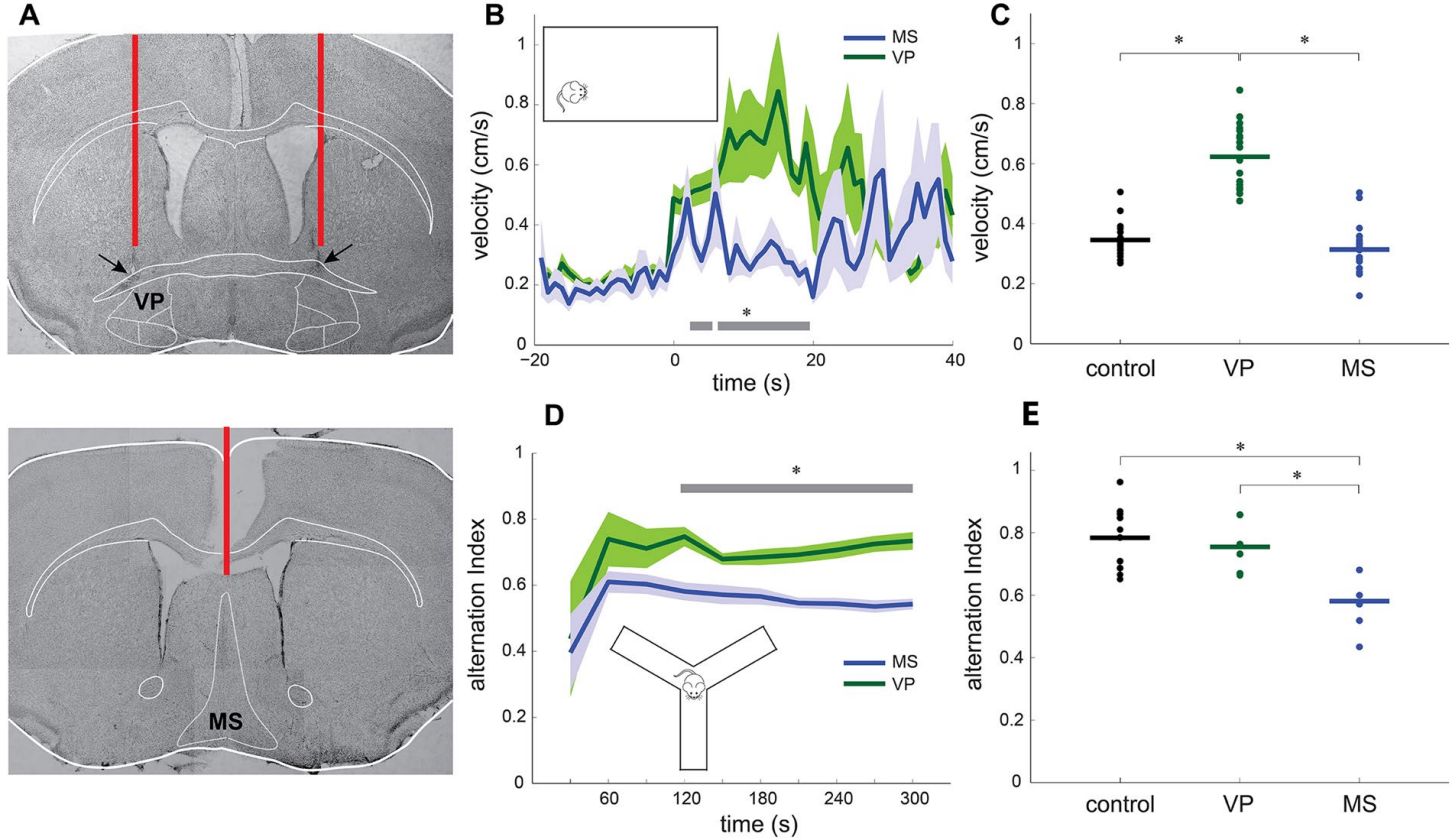
Rostral basal forebrain somatostatin cells regulate running speed and spatial working memory. (**A**) Photomontage of Nissl stained brain coronal sections from example animals (VP, NE105; MS, NE143) showing the track and final position of optic fibers chronically implanted for photostimulation in behavioral experiments. Red lines represent optic fibers. Arrows depict lesions in the basal forebrain. (**B**) Average velocity of resting transgenic mice (NpHR+) in the open field (inset) upon optogenetic stimulation of the rostral basal forebrain, in either VP (green) or MS (blue). Shading depicts standard error. Grey line shows significant differences between distributions (two-sided t-test, p < 0.05). Time reference is laser stimulation onset (single train of 10 1-s pulses delivered randomly every 2–3 minutes). (**C**) Average running speed of mice during photostimulation. Control, n = 8 NpHR- mice (4 VP-implanted, 4 MS-implanted; intra-group comparison, two-sided t-test, P = 0.35); VP, n = 5 NpHR+ mice; MS, n = 5 NpHR+ mice. One-way ANOVA, P = 1.04 × 10^−17^). Asterisks indicate significant differences (P < 0.05, pairwise Tukey’s test). (**D**) Alternation index of freely-exploring transgenic mice (NpHR+) in the Y-maze (inset) upon optogenetic stimulation of the rostral basal forebrain in either VP or MS. Shading depicts standard error. Black line shows significant differences between distributions (two-sided Wilcoxon signed-rank test, p < 0.05). Time reference is the onset of spontaneous exploration in the Y-maze (30-s blocks) during laser stimulation (5-s pulse every 20 s). (**E**) Average alternation index during Y-maze exploration sessions. Control, n = 8 NpHR- mice (4 VP-implanted, 4 MS-implanted; intra-group comparison, two-sided t-test, P = 0.3226); VP, n = 4 NpHR+ mice; MS, n = 5 NpHR+ mice. One-way ANOVA, P = 0.0028. Asterisks indicate significant differences (p < 0.05, pairwise Tukey’s test).

We found that basal forebrain pallidal somatostatin cells can regulate local gamma oscillations. In addition, we have previously shown that the same neurons can gate basal forebrain synaptic transmission and modulate cortical dynamics^26^. One possible directional mechanism of interregional crossmodulation is the functional coupling of gamma oscillations between the basal forebrain and neocortex, particularly during internally-oriented brain states^24^. We thus assessed the effect of photosuppressing somatostatin cells in simple behavioral patterns that require operational interregional cortical interactions. For this, we first tracked by video recording the locomotor activity of mice bilaterally implanted with optic fibers directed to VP or with a single fiber implanted in the midline targeting the MS (Fig. 3A). Animals were placed in an open field and allowed to freely explore the environment. During exploration we randomly photosuppressed basal forebrain somatostatin cells and measured animals’ movement speed (Fig. 3B). Locomotor displacements were significantly faster in VP-photostimulated mice than MS-photostimulated animals when somatostatin cells were transiently inactivated during quiescence (Fig. 3B). Notably, the effect was anatomically restricted as suppression of septal somatostatin cells did not affect movement speed, neither with trains (Fig. 3B) nor with prolonged pulses (two-sided t-test, P = 0.3425). Importantly, MS implanted animals performed at levels comparable to control animals, whereas VP animals were significantly enhanced (Fig. 3C). Interestingly, no optogenetic effect was detected on movement speed when animals were running at the time of stimulation (one-way ANOVA, P = 0.0928). Thus, optogenetic inactivation of pallidal somatostatin neurons selectively increased movement speed in resting animals, when the default mode network is maximally active^25^.

We then evaluated a simple cognitive task by quantifying spatial working memory in the Y-maze spontaneous alternation test, in mice implanted with optic fibers targeting either the MS or VP. Animals were placed in the Y-maze and allowed to explore freely the arms. During exploration, we delivered recurrent photostimulation to the basal forebrain and computed the number and order of arm entries to calculate the percentage of alternation, an index of spatial working memory^31^. Interestingly, spontaneous alternation was decreased in MS transgenic animals as compared to VP transgenic mice when somatostatin cells were suppressed (Fig. 3D). Remarkably, the effect was also domain-specific as implanted VP animals performed at similar levels when compared to control animals, whereas MS animals were significantly impaired (Fig. 3E). Thus, optogenetic inactivation of somatostatin neurons in different anatomical domains of the rostral basal forebrain functionally segregates distinct neural circuits for the control of motor or cognitive activity.

## Material and Methods

All procedures involving experimental animals were performed in accordance to the U.S. Public Health Service’s Policy on Humane Care and Use of Laboratory Animals, reviewed and approved by university (Comite Etico Cientifico para el cuidado de animales y ambiente, CEC-CAA) and national (Comision Nacional de Investigacion Cientifica y Tecnologica, CONICYT) bioethics committees. Experiments were carried out with 8- to 30-week-old mice (n = 27 from either sex), in accordance with the Ethics Committee (protocol CEBA 13-014).

### Animals

Three mice strains from Jackson laboratories (www.jax.org) were used in this study, C57Bl/6J (stock N° 000664), Ai39 (stock N° 014539, B6, 129S-Gt(ROSA)26Sor^tm39(CAG-HOP/EYFP)Hze^/J), and Sst-IRES-Cre (stock N° 013044, Sst^tm2.1(cre)Zjh^/J and stock N° 018973, B6N.Cg-Sst^tm2.1(cre)Zjh^/J). They were used as controls and we refer to them as (Natronomonas pharaonis halorhodopsin) NpHR- animals in the text. Double transgenic animals were obtained from the breeding of Sst-IRES-Cre and Ai39 mice, so that they expressed functional (NpHR) exclusively in somatostatin cells^26^. We refer to such animals as NpHR + in the text. Mice were genotyped by PCR on ear biopsies using the primers: GGG CCA GGA GTT AAG GAA GA (common), TCT GAA AGA CTT GCG TTT GG (wild type forward), TGG TTT GTC CAA ACT CAT CAA (mutant forward) for CRE Mice, and CTT TAA GCC TGC CCA GAA GA (wild type reverse), ATA TCC TGC TGG TGG AGT GG (mutant forward), GCC ACG ATA TCC AGG AAA GA (mutant reverse), TCC CAA AGT CGC TCT GAG (wild type forward) from Integrated DNA Technologies.

### *In vivo* electrophysiological recordings

Animals were induced with isoflurane, and then anesthetized with urethane (0.8 g/kg), and after 20 minutes a dose of ketamine (40 g/kg)/xylazine (4 g/kg) to start the surgical procedures^32^. Throughout the experiment 1/12 of the initial dose of urethane was administered every 20–30 minutes. All drugs were administered intraperitoneally. Rectal temperature was monitored throughout the experiment and was kept at 36 ± 1 °C with a heating pad. Glucosaline solution was injected subcutaneously every 2 hours. In fully anesthetized mice, the scalp was cut and retracted to expose the skull. Mice were then implanted with a customized lightweight metal head holder and the head was held in a custom-made metallic holder. Next, a small craniotomy (~1 mm) was made with a dental drill above the basal forebrain^33^, either to target the ventral pallidum (AP 0.0 mm, ML 1.5 mm, DV 4.0 mm from Bregma) or the medial septum (AP 1.0 mm, ML 0.0 mm, DV 2.5 mm from Bregma). The exposed dura was cut to expose the cortex giving access for implantation of the optic fiber and recording electrodes. Neuronal activity in basal forebrain was recorded by using either a 16 or a 32 channel-silicon probe (Neuronexus) stained with DiI and connected to an optic fiber (100 μm in diameter) attached to the shank (optrode). Electrical activity was recorded with a 32-channel Intan RHD 2132 amplifier board connected to an RHD2000 evaluation system (Intan Technologies). Single-unit activity and local field potential (LFP; sampling rate 20 kHz) were digitally filtered between 300 Hz–5 kHz and 0.3 Hz–2 kHz, respectively. Spike shape and amplitude were monitored during recording to ensure that the same cells were recorded. Electrical recordings could be potentially contaminated by non-local and non-neural activity arising from the reference electrode^34^. This is especially critical for recordings in awake behaving animals because of movement-related noise. Yet, here we present data acquired from the anesthetized preparation where some precautions were taken to control these unwanted artifacts. Thus, for all the experiments we used a metal pin implanted and cemented to the skull, while keeping away the scalp to prevent interference from muscle or skin activity. The pin was implanted at AP 2.0–2.5 mm and ML 1.5–2 mm from Bregma, i.e. on top of the primary and secondary cortical motor areas. In this way we prevented artifacts arising from multiple sources such as heartbeats or respiration. All data has been made publicly available (http://crcns.org/data-sets/bf/bf-2)

### Surgery for chronic implantation

Mice were anesthetized with isoflurane (5% induction and 1.5–2% maintenance) and placed on a stereotaxic frame (David Kopf Instruments). Temperature was kept at 37° throughout the procedure (1–2 hours) using a heating pad. The skin was incised to expose the skull and a craniotomy (~1 mm in diameter) was made with a dental drill above the basal forebrain bilaterally (coordinates as above^33^). Two optic fibers (diameter 200 um, length 10 mm; Thorlabs) were inserted and glued to ceramic ferrules (diameter 230 um, length 6 mm; Thorlabs) were descended through both craniotomies until reaching the basal forebrain and anchored to the skull using dental cement. After surgery, mice received a daily dose of enrofloxacin (10 mg/kg, Centrovet) for three days and supplementary analgesia with ketoprofen (5 mg/kg, Centrovet) for three days. Animals were allowed at least a week of recovery before behavioral tests.

### Optogenetic stimulation

Optogenetic stimulation of basal forebrain somatostatin neurons was achieved with a 200 μm optic fiber, (N.A. 0.37, Thorlabs) coupled to a green laser (532 nm) that provided a total light power of 0.1–60 mW at the fiber tip. An optrode was also used, which consisted of an optic fiber (100 μm, N.A. 0.22, Neuronexus) attached to an array of electrodes, so electrical recording and photostimulation can be achieved simultaneously on the same site. Light stimuli consisted of 5 second light pulses and power at the tip of the fiber was set at 10–15 mW for 200 μm optic fiber. Every recording session lasted 10 minutes, during which laser stimulation was continuously presented for 5 seconds every 20 seconds (i.e.; 15 sec. off – 5 sec. on). Gamma events were compared between trials of 5 sec laser on and the previous 5 sec laser off periods. The reason for this window was to prevent potential contamination by changes in neural activity extending after the end of optical stimulation.

For chronically implanted animals, we randomly delivered a pulse train (10 consecutive square pulses at 0.5 Hz [1 s on, 1 s off], 15–20 mW) every 1–3 min.

Identification of cell types through photoinhibition is difficult to prove as spiking suppression could occur through multi-synaptic processes instead of direct inhibition. For this, we measured the latency of photoinhibition upon laser stimulation to confirm the functional expression of NpHR in somatostatin cells (Supplementary Fig. 2).

### Open field test

The testing arena (50 cm × 30 cm × 30 cm vertical) was made of black painted acrylic, and illuminated by a 60 W bulb placed 150 cm above. The bilaterally implanted animal was connected to optical fibers (200 um diameter, 1 m length) and placed on its cage to be habituated to the room for at least 15 min before testing. Next, the animal was placed in the testing arena for 10 minutes with no laser stimulation. This procedure was performed for three days to habituate the animal both to the room and arena. On the fourth day, the animal was placed in the testing arena for one hour before starting the stimulation protocol. The test was recorded using a digital video camera with a frame rate of 30 FPS. In some experiments, light transmission through cannulae was blocked with a small piece of aluminum foil placed between the ferrules.

### Y-maze

Short-term spatial working memory was tested by recording spontaneous alternation behavior in the Y-maze. Mice were placed at the end of one arm and allowed to explore the three arms freely for 5 min. Three consecutive choices of all three arms were regarded as a correct alternation. The percentage of spontaneous alternation was calculated by dividing the total number of alternations by the total number of choices minus 2 and then multiplying by 100. Movements were recorded with a video camera and analyzed using Idtracker^35^.

### Histology

At the end of recordings, mice were terminally anesthetized and intracardially perfused with saline followed by 20 min fixation with 4% paraformaldehyde. Brains were extracted and postfixed in paraformaldehyde for a minimum of 8 h before being transferred to PBS azide and sectioned coronally (60–70 μm slice thickness). Sections were further stained for Nissl substance. Location of shanks and optical fiber were determined in reference to standard brain atlas coordinates^33^ under a light transmission microscope (Supplementary Table 3). For immunocytochemistry, non-recorded NpHR+ animals were terminally anesthetized and intracardially perfused with saline followed by 20 min fixation with 4% paraformaldehyde. Brains were extracted and sectioned coronally (60–70 μm slice thickness). Sections were heated at 80 °C for 10 minutes in citric acid (pH 6.0), after cooling, sections were rinsed three times for 10 min each with phosphate buffer, incubated in 1% horse serum supplemented with 0.3% Triton X-100 in phosphate buffer for 1 h, and then incubated in 1:1000 dilution of somatostatin antibody (Abcam, ab64053) for 24 h at 4 °C, followed by a 1:500 dilution of the secondary antibody for 3–6 hours at room temperature. Secondary antibodies were conjugated to Alexa Fluor 568 (Invitrogen); and cells were photographed with the appropriate filter cubes (Nikon; B-2E-C for EYFP, and G-2E/C for Alexa) with an epifluorescence microscope (Supplementary Fig. 3). Antibody dilutions were performed in phosphate buffer with 1% horse serum and 0.3% Triton X-100. Sections were mounted on slides with mounting medium and photographed under epiluminescence microscopy.

### Spike sorting

Semiautomatic clustering was performed by KlustaKwik, a custom program written in C++ (https://github.com/kwikteam/klustakwik2/). This method was applied over the 32 channels of the silicon probe, grouped in eight pseudo-tetrodes of four nearby channels. Spike clusters were considered single units if their auto-correlograms had a 2-ms refractory period and their cross-correlograms with other clusters did not have sharp peaks within 2 ms of 0 lag.

### Unit cross-correlation analysis

Neural activity in the cortex and basal forebrain was cross-correlated with the light pulse by applying the “sliding-sweeps” algorithm^36^. A time window of ±15 s was defined with point 0 assigned to the light onset. The timestamps of the cortical and basal forebrain spikes within the time window were considered as a template and were represented by a vector of spikes relative to t = 0 s, with a time bin of 500 ms and normalized to the basal firing rate of the neurons. Thus, the central bin of the vector contained the ratio between the number of neural spikes elicited between ± 250 ms and the total number of spikes within the template. Next, the window was shifted to successive light pulses throughout the recording session, and an array of recurrences of templates was obtained. Both neural timestamps and start times of light pulses where shuffled by randomized exchange of the original inter-event intervals and the cross-correlation procedure was performed on the random sequence. The statistical significance of the observed repetition of spike sequences was assessed by comparing, bin to bin, the original sequence with the shuffled sequence. An original correlation sequence that presented a statistical distribution different from 100 permutations was considered as statistically significant, with p < 0.01 probability, instead of a chance occurrence.

### Spectral analysis

Time-frequency decomposition of LFP was performed with multi-taper Fourier analysis^37^ implemented in Chronux toolbox (http://www.chronux.org). LFP was downsampled to 500 Hz before decomposition. The same taper parameters described for the coherence analysis were used. To estimate gamma band power, spectra were normalized by 1/f^37^, in order to correct for the power law governing the distribution of EEG signals. To compute power and frequency of the gamma band oscillation, LFP was band-pass filtered with a two-way least squares FIR filter (eegfilt.m from EEGLAB toolbox; http://www.sccn.ucsd.edu/eeglab/).

### Single-unit vs multi-unit coherence analysis

Single unit versus multi-unit coherence was determined using a previously described method^7^. Briefly, multi-unit activity was defined as the summed activity of all simultaneously recorded single units except the single unit used as reference for comparison. Spiking activity was then binned at 500 Hz and coherence for each single unit versus multi-unit pair was averaged for light ON (5 s) and light OFF (5 s before light onset) epochs. Coherence was computed using the multi-taper Fourier analysis^37^ as implemented in the Chronux toolbox (http://www.chronux.org). For each 5 s epoch, coherence was calculated using a time-bandwidth product of TW = 3 and 2TW-1 = 5 tapers, resulting in a half bandwidth W = 0.6 Hz. Wilcoxon signed rank test was applied to estimate the statistical significance of coherence results.

### Detection of gamma band oscillations

A method developed by Logothetis *et al*.^38^ to detect fast oscillations in the hippocampus was modified to detect gamma waves. Briefly, cortical local field potential was downsampled (500 Hz) and band-pass filtered (20–80 Hz) using a zero-phase shift non-causal finite impulse filter with 0.5 Hz roll-off. Next, the signal was rectified, and low pass filtered at 20 Hz with a 4th order Butterworth filter. This procedure yields a smooth envelope of the filtered signal, which was then z-score normalized using the mean and SD of the whole signal. Epochs during which the normalized signal exceeded a 2 SD threshold were considered as events. The first point before threshold that reached 1 SD was considered the onset and the first one after threshold to reach 1 SD as the end of events. The difference between onset and end of events was used to estimate the gamma duration. We introduced a 150 ms-refractory window to prevent double detections. In order to precisely determine the mean frequency, amplitude, and duration of each event, we performed a spectral analysis using Morlet complex wavelets of seven cycles. Finally, a minimum duration criterion of 150 ms was used. The Matlab toolbox used is available online as LAN-toolbox (https://bitbucket.org/marcelostockle/lan-toolbox/wiki/Home).

### Statistics

Data sets were tested for normality using Kolmogorov-Smirnov test and then compared with the appropriate test (t-test or Wilcoxon two-sided rank test). Statistical significance of data for protocols with factorial design (i.e., light on/off conditions) were assessed using two-way repeated-measures ANOVA followed by false discovery rate (FDR) for multiple comparison corrections or Kruskal Wallis test followed by a by Mann-Whitney U contrasts.

## Discussion

Gamma rhythms are network oscillations present in many brain regions across all states of vigilance, yet their functions and mechanisms remain unclear^39^. Gamma oscillations seem to be important for efficient cortical cognitive operations^40–42^, including memory^43,44^, attention^21^, cognitive flexibility^45^ and sensory processing^20^. It is now known that specific cell types, particularly GABAergic cells, are of relevance for the generation and coordination of gamma oscillations^20,39,46^. Over the last few years increasing evidence has shown the importance of somatostatin cells in regulating neuronal activity across the brain, including a prominent role in the coordination of gamma oscillations. Indeed, somatostatin cells modulate gamma oscillations and odor discrimination in the olfactory bulb^47^. In the hippocampus, bistratified cells, which express high levels of somatostatin in their somata, are tightly coupled to spontaneous gamma oscillations^48^, which frequency defines either internal coupling (i.e.; CA3 to CA1) or external interregional coordination (i.e.; entorhinal cortex to CA1)^49^. In the visual cortex, somatostatin neurons synchronize distributed networks through gamma band oscillations^46^. These oscillations also organize descending cortical signaling to the hypothalamus enabling food-seeking behavior, and the impinging gamma-rhythmic input arises from lateral septum somatostatin cells^50^. Finally, we have demonstrated the role of basal forebrain somatostatin cells in the indirect regulation of gamma oscillations in the prefrontal cortex^26^. Even though GABAergic somatostatin cells do not comprise a distinct cell type common to different brain structures, compelling evidence has functionally linked their activity to the organization of gamma oscillations across different brain regions. Nevertheless, such functional connection has not yet been established for the generation and coordination of gamma oscillations in the basal forebrain.

Basal forebrain somatostatin cells comprise a small, yet diverse neuronal population. Indeed, in the regulation of the sleep-wake cycle, somatostatin cells exert a unique effect in promoting sleep, even though many of them are maximally activated during wakefulness^5^. Also interesting is the fact that optogenetic activation of basal forebrain pallidal somatostatin neurons during wakefulness increases high-calorie food intake and anxiety-like behaviors^51^, suggesting specialization of different subsets of somatostatin cells in distinct functional roles. On the other hand, recent evidence has causally connected basal forebrain gamma oscillations with the regulation of the default mode network^24^, suggesting that the basal forebrain can act as a node commanding transitions between internally and externally oriented brain states. One of the main functional hubs of the default mode network is the medial prefrontal cortex^25^, which is synaptically targeted and strongly influenced by the ventral pallidum in the basal forebrain^52,53^. A recent study established that one branch of the basal forebrain recruits the prefrontal cortex into the default mode network by means of directional gamma oscillations^24^, making it significant to establish their synaptic origin. Here, we found that somatostatin cells regulate local gamma oscillations in the ventral pallidum, but that action is not extensive to the medial septum, even though these are neighboring regions in the rostral basal forebrain^54^. This result might be surprising, as both basal forebrain regions express prominent gamma oscillations. They both contain similar neuronal populations, including cholinergic cells, parvalbumin-expressing GABAergic cells, glutamatergic cells and somatostatin cells^13^, which provide long-range projections that widely reach cortical and subcortical targets^17,55,56^. Nonetheless, afferent and efferent connectivity is entirely different for both domains. The ventral pallidum projects prominently to the prefrontal cortex^56^, whereas the medial septum targets the hippocampal formation^57^. This could be related with a differential origin of gamma oscillations. Indeed, the hippocampus displays prominent theta-modulated gamma oscillations^39^, which could be directly transmitted to the medial septum by long-range projection fibers. In fact, a small population of somatostatin GABAergic cells in the CA1 stratum oriens directly targets the medial septum delivering theta-modulated inputs, which are also strongly driven by sharp wave ripples^58^. Thus, the synaptic origin of septal gamma oscillations may be located distally in the hippocampus, which is also consistent with our observation of little gamma-modulation in septal neuronal discharge. However, future experiments will have to be performed to test this idea. In addition, we have previously shown that somatostatin cells indirectly upregulate cortical gamma oscillations, likely by disinhibiting the long-range projection neurons from the ventral pallidum^26^. Conversely, our current results propose that somatostatin neurons exert a direct role in gamma oscillations locally in the basal forebrain, which is why the rhythm decreases upon photosuppression. This result is not incompatible with the previously reported cortical effect. Instead, they complement each other. Indeed, local suppression of gamma oscillations in the ventral pallidum correlates with cortical enhancement of gamma oscillations. This is also consistent with the local synaptic connectivity in the basal forebrain, where somatostatin cells provide synaptic inhibition to the main cell types; that is, parvalbumin cells, cholinergic cells and glutamatergic cells^5^.

One potential limitation of our study is the fact that both lateral and medial septum contain somatostatin cells^13,17^, yet they contribute to different neural circuits. Despite the fact that both lateral and medial septum receive descending afferents from the hippocampus^59^, their output connectivity is largely divergent. Indeed, the medial septum yields ascending projections targeting essentially the hippocampal formation and associated cortex (i.e; entorhinal, perirhinal, and retrosplenial cortex)^60^; whereas the lateral septum outputs descending fibers mostly innervating the hypothalamus^61^. As a result, optogenetic inactivation of somatostatin cells in the septum is likely to produce mixed physiological and behavioral effects by the partial inactivation in both septal domains. The hypothalamus regulates innate behaviors such as aggression, feeding, and running^62^. Interestingly, our optogenetic experiments did not show changes in running speed when the septum was targeted by laser stimulation, which could be reasonably expected if hypothalamic synaptic targets were to be affected. Instead, septal optical stimulation disrupted spatial working memory, a cognitive function that relies on the preserved functionality of the hippocampus^31,43,63^, a prominent target of the medial septum. Although we cannot rule out in our experiments an optogenetic effect on lateral septal somatostatin cells, the main behavioral outcome seems to be chiefly mediated by activity changes in the medial septum. Conversely, optogenetic inhibition of somatostatin cells in the ventral pallidum disrupted running speed and not spatial working memory, as could be expected from the extensive pallidal innervation of the prefrontal cortex^2^ that powerfully modulates its neural dynamics^26^. Importantly, the prefrontal cortex may not always be necessary for alternation performance in rodents. In fact, some studies have not detected manifest effects of prefrontal lesions on alternation behavior^64,65^. Instead, it has been argued that prefrontal cortex may be important when delays are introduced between the sample and choice trial^66–69^, which is not the case of our experiments. Thus, the prefrontal cortex and hippocampus may be playing different roles in spatial alternation^70^, consistent with our contrasting results.

Another important factor to consider the scope and implication of our results is the fact that we performed experiments under anesthesia. Previous studies have claimed that some forms of anesthesia can be, at least partially, assimilated to sleep^71^, since they preserve physiological cortical rhythms such as slow waves^72^ or thalamic spindles^73^. In this sense, urethane anesthesia has been extensively used as it also reproduces cortical oscillations characteristic of activated states, such as theta and gamma oscillations^48,74^. Gamma oscillations are typically fast (around 60 Hz) in the awake state, but significantly slowed down (around 30 Hz) under anesthesia^75^. We found similar results in the basal forebrain, where awake gamma oscillations are rather fast (around 60 Hz^24^), yet exhibit a slower range under urethane anesthesia (around 30 Hz). Regarding functional connectivity, there are inconsistent reports about the effect of anesthesia on large-scale networks, like the default mode network^76,77^. A recent study suggests that urethane is one of the few anesthetic regimes that preserves a functional brain connectivity matrix that resembles the awake state^78^. Another issue of significance under anesthesia is the functional diversity of somatostatin cells, which has been evidenced across the sleep-wake cycle^5^. It is expected that at least a fraction of somatostatin cells will be silent under anesthesia and will thus not be detected by optogenetic suppression. Those cells could be potentially active during the behavioral tests in the waking state. As a result, it is plausible that we underestimate the proportion of somatostatin cells and their impact on the network. To properly test this condition, future experiments in chronically implanted animals will have to be performed. Overall, results from anesthesia cannot be easily or directly translated into physiological brain states such as sleep or wakefulness. Nevertheless, our results can provide some mechanistic insight into how basal forebrain circuits operate under *in vivo* conditions.

Also interesting was the observation of running speed modulation upon optogenetic stimulation of the ventral pallidum. The septohippocampal cholinergic pathway is known to strongly regulate hippocampal theta oscillations^79^, which frequency controls running speed in mice^80^. This operates in parallel to the glutamatergic pathway that also regulates locomotion and theta oscillations^81^. Thus, it would be reasonable to expect that disrupting neuronal activity in that circuit would affect locomotor speed. Instead, we found that ventral pallidal stimulation generated a noticeable effect in locomotor patterns. We have previously shown that the prefrontal cortex is significantly activated when ventral pallidal somatostatin cells are optogenetically silenced^26^; thus, one possible explanation is that the primary and secondary motor cortex is directly excited by the impinging afferents from the prefrontal cortex^82^, resembling previous experiments where indiscriminate optogenetic stimulation of pyramidal cells in the motor cortex dramatically increases running speed in mice^83^. Another, non-exclusive possibility is disinhibition of the cholinergic corticopetal pathway from the ventral pallidum to the temporal cortex, that includes the motor cortex^17^. There are very little direct projections from basal forebrain somatostatin cells to the temporal cortex^17^, but there is a significant amount targeting the basal ganglia^17^, which could also be related to the behavioral effect here described. Moreover, pallidal somatostatin cells project directly to the medial septum^17^, suggesting crossregional modulation of activity between these regions, which was not manifestly exposed in our experiments. Finally, the suppression of gamma oscillations by photoinactivation of pallidal somatostatin cells is consistent with the functional disruption of the default mode network in quiescent animals^24^.

Given their similar synaptic organization and location in the rostral basal forebrain, we expected that effects produced by ventral pallidum and medial septum might be comparable and mediated by similar mechanisms; specifically, by the directional action of gamma oscillations, as proposed for the recruitment of the default mode network^24^. Nevertheless, we found that optogenetic silencing of somatostatin cells differentially affected gamma oscillations in the basal forebrain, with distinct behavioral outcomes. As discussed, our results suggest that although neighboring rostral basal forebrain structures contain similar cell types projecting to cortical targets, they may utilize different communication channels (i.e.; gamma oscillations) to differentially modulate behavior (e.g.; motor or cognitive outputs). In summary, by using optogenetic stimulation in transgenic mice we have shown that somatostatin cells are differentially correlated with gamma band activity in the rostral basal forebrain. Pallidal somatostatin cells were synchronized with gamma band oscillations, whereas septal somatostatin cells were uncoupled to the gamma rhythm. Furthermore, optogenetic inactivation of somatostatin cells disrupted gamma waves only in the ventral pallidum, without noticeable effects in the medial septum, suggesting a causal link for somatostatin cells in the coordination of gamma oscillations in the ventral pallidum only. Finally, anatomically-selective inhibition of somatostatin cells exerted distinctive behavioral effects, with the ventral pallidum regulating locomotor speed, possibly mediated by the directional action of gamma oscillations on the default mode network, and the septal complex modulating spatial working memory. Overall, our study identifies somatostatin cells as a circuit element that differentially enables gamma synchronization to guide the activity of subcortical networks and regulate behavior by the dynamic reorganization of functional neuronal circuits.

## Supplementary information

Supplementary Dataset 1

## References

[1] Jones BE (2008). Modulation of cortical activation and behavioral arousal by cholinergic and orexinergic systems. Annals of the New York Academy of Sciences.

[2] Zaborszky, L., Van den Pol A. & Gyengesi E. The Basal Forebrain Cholinergic Projection System in Mice. *The Mouse Nervous System*, (Elsevier Inc.), pp 684–718 (2012).

[3] Lin SC, Brown RE, Hussain Shuler MG, Petersen CC, Kepecs A (2015). Optogenetic Dissection of the Basal Forebrain Neuromodulatory Control of Cortical Activation, Plasticity, and Cognition. The Journal of neuroscience: the official journal of the Society for Neuroscience.

[4] Kilgard MP, Merzenich MM (1998). Cortical map reorganization enabled by nucleus basalis activity. Science.

[5] Xu M (2015). Basal forebrain circuit for sleep-wake control. Nature neuroscience.

[6] Froemke RC, Merzenich MM, Schreiner CE (2007). A synaptic memory trace for cortical receptive field plasticity. Nature.

[7] Pinto L (2013). Fast modulation of visual perception by basal forebrain cholinergic neurons. Nature neuroscience.

[8] Moruzzi G, Magoun HW (1949). Brain stem reticular formation and activation of the EEG. Electroencephalography and clinical neurophysiology.

[9] Blanco-Centurion C, Gerashchenko D, Shiromani PJ (2007). Effects of saporin-induced lesions of three arousal populations on daily levels of sleep and wake. The Journal of neuroscience: the official journal of the Society for Neuroscience.

[10] Whitehouse PJ (1982). Alzheimer’s disease and senile dementia: loss of neurons in the basal forebrain. Science.

[11] Conner JM, Culberson A, Packowski C, Chiba AA, Tuszynski MH (2003). Lesions of the Basal forebrain cholinergic system impair task acquisition and abolish cortical plasticity associated with motor skill learning. Neuron.

[12] Smith JE (2004). Involvement of cholinergic neuronal systems in intravenous cocaine self-administration. Neuroscience and biobehavioral reviews.

[13] Zaborszky L, Duque A (2000). Local synaptic connections of basal forebrain neurons. Behavioural brain research.

[14] Jones BE (2005). From waking to sleeping: neuronal and chemical substrates. Trends in pharmacological sciences.

[15] Brashear HR, Zaborszky L, Heimer L (1986). Distribution of GABAergic and cholinergic neurons in the rat diagonal band. Neuroscience.

[16] Boucetta S, Cisse Y, Mainville L, Morales M, Jones BE (2014). Discharge profiles across the sleep-waking cycle of identified cholinergic, GABAergic, and glutamatergic neurons in the pontomesencephalic tegmentum of the rat. The Journal of neuroscience: the official journal of the Society for Neuroscience.

[17] Do JP. *et al*. Cell type-specific long-range connections of basal forebrain circuit. *eLife* 5 (2016). 10.7554/eLife.13214 PMC5095704 27642784

[18] Hu H, Gan J, Jonas P (2014). Interneurons. Fast-spiking, parvalbumin(+) GABAergic interneurons: from cellular design to microcircuit function. Science.

[19] Halassa MM (2014). State-dependent architecture of thalamic reticular subnetworks. Cell.

[20] Cardin JA (2009). Driving fast-spiking cells induces gamma rhythm and controls sensory responses. Nature.

[21] Kim H, Ahrlund-Richter S, Wang X, Deisseroth K, Carlen M (2016). Prefrontal Parvalbumin Neurons in Control of Attention. Cell.

[22] Kim T (2015). Cortically projecting basal forebrain parvalbumin neurons regulate cortical gamma band oscillations. Proceedings of the National Academy of Sciences of the United States of America.

[23] Sohal VS, Zhang F, Yizhar O, Deisseroth K (2009). Parvalbumin neurons and gamma rhythms enhance cortical circuit performance. Nature.

[24] Nair J (2018). Basal forebrain contributes to default mode network regulation. Proceedings of the National Academy of Sciences of the United States of America.

[25] Gusnard DA, Akbudak E, Shulman GL, Raichle ME (2001). Medial prefrontal cortex and self-referential mental activity: relation to a default mode of brain function. Proceedings of the National Academy of Sciences of the United States of America.

[26] Espinosa N. *et al*. Basal Forebrain Gating by Somatostatin Neurons Drives Prefrontal Cortical Activity. *Cerebral cortex*:1–12 (2017). 10.1093/cercor/bhx302 29161383

[27] Le Van Quyen M (2010). Large-scale microelectrode recordings of high-frequency gamma oscillations in human cortex during sleep. The Journal of neuroscience: the official journal of the Society for Neuroscience.

[28] Sirota A (2008). Entrainment of neocortical neurons and gamma oscillations by the hippocampal theta rhythm. Neuron.

[29] Valderrama M (2012). Human gamma oscillations during slow wave sleep. PloS one.

[30] Vinck M, van Wingerden M, Womelsdorf T, Fries P, Pennartz CM (2010). The pairwise phase consistency: a bias-free measure of rhythmic neuronal synchronization. NeuroImage.

[31] Sarnyai Z (2000). Impaired hippocampal-dependent learning and functional abnormalities in the hippocampus in mice lacking serotonin(1A) receptors. Proceedings of the National Academy of Sciences of the United States of America.

[32] Negron-Oyarzo I, Neira D, Espinosa N, Fuentealba P, Aboitiz F (2015). Prenatal Stress Produces Persistence of Remote Memory and Disrupts Functional Connectivity in the Hippocampal-Prefrontal Cortex Axis. Cerebral cortex.

[33] Franklin, K. B. J. & Paxinos, G. *The Mouse Brain in Stereotaxic Coordinates* (Academic Press) (2007).

[34] Whitmore NW, Lin SC (2016). Unmasking local activity within local field potentials (LFPs) by removing distal electrical signals using independent component analysis. Neuroimage.

[35] Perez-Escudero A, Vicente-Page J, Hinz RC, Arganda S, de Polavieja GG (2014). idTracker: tracking individuals in a group by automatic identification of unmarked animals. Nature methods.

[36] Abeles M, Gerstein GL (1988). Detecting spatiotemporal firing patterns among simultaneously recorded single neurons. Journal of neurophysiology.

[37] Mitra PP, Pesaran B (1999). Analysis of dynamic brain imaging data. Biophys J.

[38] Logothetis NK (2012). Hippocampal-cortical interaction during periods of subcortical silence. Nature.

[39] Buzsaki G, Wang XJ (2012). Mechanisms of gamma oscillations. Annual review of neuroscience.

[40] Gray CM, Singer W (1989). Stimulus-specific neuronal oscillations in orientation columns of cat visual cortex. Proceedings of the National Academy of Sciences of the United States of America.

[41] Salinas E, Sejnowski TJ (2001). Correlated neuronal activity and the flow of neural information. Nature reviews. Neuroscience.

[42] Csicsvari J, Jamieson B, Wise KD, Buzsaki G (2003). Mechanisms of gamma oscillations in the hippocampus of the behaving rat. Neuron.

[43] Yamamoto J, Suh J, Takeuchi D, Tonegawa S (2014). Successful execution of working memory linked to synchronized high-frequency gamma oscillations. Cell.

[44] Igarashi KM, Lu L, Colgin LL, Moser MB, Moser EI (2014). Coordination of entorhinal-hippocampal ensemble activity during associative learning. Nature.

[45] Cho KK (2015). Gamma rhythms link prefrontal interneuron dysfunction with cognitive inflexibility in Dlx5/6(+/−) mice. Neuron.

[46] Veit J, Hakim R, Jadi MP, Sejnowski TJ, Adesnik H (2017). Cortical gamma band synchronization through somatostatin interneurons. Nature neuroscience.

[47] Lepousez G, Mouret A, Loudes C, Epelbaum J, Viollet C (2010). Somatostatin contributes to *in vivo* gamma oscillation modulation and odor discrimination in the olfactory bulb. The Journal of neuroscience: the official journal of the Society for Neuroscience.

[48] Tukker JJ, Fuentealba P, Hartwich K, Somogyi P, Klausberger T (2007). Cell type-specific tuning of hippocampal interneuron firing during gamma oscillations *in vivo*. The Journal of neuroscience: the official journal of the Society for Neuroscience.

[49] Colgin LL (2009). Frequency of gamma oscillations routes flow of information in the hippocampus. Nature.

[50] Carus-Cadavieco M (2017). Gamma oscillations organize top-down signalling to hypothalamus and enable food seeking. Nature.

[51] Zhu C (2017). Somatostatin Neurons in the Basal Forebrain Promote High-Calorie Food Intake. Cell reports.

[52] Bloem B (2014). Topographic mapping between basal forebrain cholinergic neurons and the medial prefrontal cortex in mice. The Journal of neuroscience: the official journal of the Society for Neuroscience.

[53] Chandler DJ, Lamperski CS, Waterhouse BD (2013). Identification and distribution of projections from monoaminergic and cholinergic nuclei to functionally differentiated subregions of prefrontal cortex. Brain research.

[54] Paxinos G *The rat nervous system* third Ed (2004).

[55] Zaborszky L (2015). Neurons in the basal forebrain project to the cortex in a complex topographic organization that reflects corticocortical connectivity patterns: an experimental study based on retrograde tracing and 3D reconstruction. Cerebral cortex.

[56] Henny P, Jones BE (2008). Projections from basal forebrain to prefrontal cortex comprise cholinergic, GABAergic and glutamatergic inputs to pyramidal cells or interneurons. The European journal of neuroscience.

[57] Kasa P (1986). The cholinergic systems in brain and spinal cord. Progress in neurobiology.

[58] Jinno S (2007). Neuronal diversity in GABAergic long-range projections from the hippocampus. The Journal of neuroscience: the official journal of the Society for Neuroscience.

[59] Alonso A, Kohler C (1982). Evidence for separate projections of hippocampal pyramidal and non-pyramidal neurons to different parts of the septum in the rat brain. Neuroscience letters.

[60] Swanson LW (1977). The anatomical organization of septo-hippocampal projections. Ciba Foundation symposium.

[61] Risold PY, Swanson LW (1997). Connections of the rat lateral septal complex. Brain research. Brain research reviews.

[62] Remedios R (2017). Social behaviour shapes hypothalamic neural ensemble representations of conspecific sex. Nature.

[63] Suh J, Rivest AJ, Nakashiba T, Tominaga T, Tonegawa S (2011). Entorhinal cortex layer III input to the hippocampus is crucial for temporal association memory. Science.

[64] Aggleton JP, Neave N, Nagle S, Hunt PR (1995). A comparison of the effects of anterior thalamic, mamillary body and fornix lesions on reinforced spatial alternation. Behavioural brain research.

[65] Deacon RM, Penny C, Rawlins JN (2003). Effects of medial prefrontal cortex cytotoxic lesions in mice. Behavioural brain research.

[66] Seamans JK, Floresco SB, Phillips AG (1995). Functional differences between the prelimbic and anterior cingulate regions of the rat prefrontal cortex. Behavioral neuroscience.

[67] Floresco SB, Seamans JK, Phillips AG (1997). Selective roles for hippocampal, prefrontal cortical, and ventral striatal circuits in radial-arm maze tasks with or without a delay. The Journal of neuroscience: the official journal of the Society for Neuroscience.

[68] Taylor CL, Latimer MP, Winn P (2003). Impaired delayed spatial win-shift behaviour on the eight arm radial maze following excitotoxic lesions of the medial prefrontal cortex in the rat. Behavioural brain research.

[69] Di Pietro NC, Black YD, Green-Jordan K, Eichenbaum HB, Kantak KM (2004). Complementary tasks to measure working memory in distinct prefrontal cortex subregions in rats. Behavioral neuroscience.

[70] Sanderson DJ, Bannerman DM (2012). The role of habituation in hippocampus-dependent spatial working memory tasks: evidence from GluA1 AMPA receptor subunit knockout mice. Hippocampus.

[71] Clement EA (2008). Cyclic and sleep-like spontaneous alternations of brain state under urethane anaesthesia. PLoS One.

[72] Steriade M, Nunez A, Amzica F (1993). A novel slow (<1 Hz) oscillation of neocortical neurons *in vivo*: depolarizing and hyperpolarizing components. The Journal of neuroscience: the official journal of the Society for Neuroscience.

[73] Steriade M (2006). Grouping of brain rhythms in corticothalamic systems. Neuroscience.

[74] Klausberger T (2003). Brain-state- and cell-type-specific firing of hippocampal interneurons *in vivo*. Nature.

[75] Xing D (2012). Stochastic generation of gamma-band activity in primary visual cortex of awake and anesthetized monkeys. J Neurosci.

[76] Greicius MD (2008). Persistent default-mode network connectivity during light sedation. Hum Brain Mapp.

[77] Deshpande G, Kerssens C, Sebel PS, Hu X (2010). Altered local coherence in the default mode network due to sevoflurane anesthesia. Brain Res.

[78] Paasonen J, Stenroos P, Salo RA, Kiviniemi V, Grohn O (2018). Functional connectivity under six anesthesia protocols and the awake condition in rat brain. Neuroimage.

[79] Buzsaki G (2002). Theta oscillations in the hippocampus. Neuron.

[80] Bender F (2015). Theta oscillations regulate the speed of locomotion via a hippocampus to lateral septum pathway. Nature communications.

[81] Fuhrmann F (2015). Locomotion, Theta Oscillations, and the Speed-Correlated Firing of Hippocampal Neurons Are Controlled by a Medial Septal Glutamatergic Circuit. Neuron.

[82] Euston DR, Gruber AJ, McNaughton BL (2012). The role of medial prefrontal cortex in memory and decision making. Neuron.

[83] Gradinaru V (2007). Targeting and readout strategies for fast optical neural control *in vitro* and *in vivo*. The Journal of neuroscience: the official journal of the Society for Neuroscience.

