# Supplementary material for: Basal forebrain somatostatin cells differentially regulate local gamma oscillations and functionally segregate motor and cognitive circuits
Source: Sci Rep. 2019 Feb 22;9:2570. doi: 10.1038/s41598-019-39203-4 (PMC6384953; doi:10.1038/s41598-019-39203-4)
Supplement: Supplementary file 1 — Supplementary Dataset 1 [file 41598_2019_39203_MOESM1_ESM.pdf]

Supplementary Material

Basal forebrain somatostatin cells differentially regulate local gamma oscillations and functionally segregate motor and cognitive circuits

Nelson Espinosa, Alejandra Alonso, Ariel Lara-Vasquez, Pablo Fuentealba

Supplementary Table 1. Contribution of individual animals to neuronal sample recorded under anesthesia

Ventral Pallidum

			laser effect		
**animal ID**	**neurons**	**sessions**	**excited**	**inhibited**	**unaffected**
NE48	36	1	10	2	24
NE52	145	8	29	13	103
NE58	97	7	10	9	78
NE69	52	4	7	5	40
total	**330**	**20**	56	29	245
%			17	8.8	74.2

Medial Septum

			laser effect		
**animal ID**	**neurons**	**sessions**	**excited**	**inhibited**	**unaffected**
NE112	6	1	1	1	4
NE113	54	4	16	4	34
NE114	62	5	12	2	48
NE115	33	3	8	5	20
NE123	1	1	0	0	1
NE124	14	2	1	1	12
NE125	7	2	0	0	7
total	**177**	**18**	38	13	126
%			21.5	7.3	71.2

Supplementary Table 2. Comparison of gamma frequency oscillations in the rostral basal forebrain.

Parameter	Medial Septum	Ventral Pallidum	P	Statistical test
Bandwidth (Hz)	12.3 ± 1.6	13.3 ± 2.5	0.717	two-sided t-test
Central Frequency (Hz)	26.3 (25.4 - 29.1)	28.6 (27.4 - 32.0)	0.0589	Wilcoxon rank-sum test
Density (Hz)	0.195 ± 0.012	0.316 ± 0.020	4.9E-6	two-sided t-test
Power (a.u.)	0.086 ± 0.019	0.022 ± 0.003	0.0084	two-sided t-test

Values are in mean ± SEM or median (interquartile range)

Supplementary Table 3: Anatomical location of chronically implanted optical fibers.

Ventral Pallidum

NpHR+	LEFT HEMISPHERE						RIGHT HEMISPHERE					TEST	
TAG	ID	SECTION	AP	ML	DV	NUCLEI	SECTION	AP	ML	V	NUCLEI	Y-maze	Open field
1189	NE99	38	-0.1	1.46	4.17	VP, SI, HDB, MCPO	32	0.1	1.1	4.4	VP, SI, HDB	*	*
1190	NE97	36	0.5	0.83	3.96	VP, SI, HDB, MCPO	36	0.5	0.8	4	VP, SI, HDB, MCPO	*	*
1191	NE98	-	-	-	-	-	-	-	-	-	-	*	*
1337	NE109	16	0.5	1.25	4.17	VP, SI, HDB, MCPO	18	0.4	1.4	4	VP, SI, MCPO	X	*
1556	NE118	17	0.38	1.46	4.38	VP, MCPO	17	0.4	0.9	4.2	VP, SI, HDB, MCPO	*	*

NpHR-	LEFT HEMISPHERE						RIGHT HEMISPHERE					TEST	
TAG	ID	SECTION	AP	ML	DV	NUCLEI	SECTION	AP	ML	V	NUCLEI	Y-maze	Open field
1289	NE107	-	-	-	-	-	-	-	-	-	-	*	*
1290	NE108	37	0.26	1.56	3.96	VP, SI, HDB, MCPO	43	0.1	1.3	4.3	VP, SI, HDB, MCPO	*	*
1335	NE105	32	0.14	1.67	4.48	VP, SI, HDB, MCPO	32	0.1	1.3	4.2	VP, SI, HDB, MCPO	*	*
1336	NE104	28	0.14	1.67	4.58	VP, SI, HDB, MCPO	27	0.1	1.3	4.4	VP, SI, HDB, MCPO	X	*
1475	NE116	33	0.74	1	4.35	VP, SI, HDB	37	0.6	1.2	4.4	VP,HDB	*	*

Medial Septum

NpHR+						TEST	
**TAG**	**ID**	**SECTION**	**AP**	**ML**	**DV**	**Y-maze**	**Open field**
1855	NE141	22	1.1	0	2.25	*	*
1856	NE144	-	-	-	-	*	X
1857	NE143	38	0.86	0	2.1	*	*
1870	NE142	17	1.1	0	2.3	X	*
1898	NE147	35	0.62	0	2.5	*	*
1899	NE148	39	0.5	0	1.8	*	*

NpHR-						TEST	
**TAG**	**ID**	**SECTION**	**AP**	**ML**	**DV**	**Y-maze**	**Open field**
1791	NE132	30	0.74	0	2.25	*	*
1792	NE131	32	0.86	0	2.2	*	*
1793	NE135	16	1.18	0	2.25	*	*
1794	NE134	24	1.1	0	2.3	*	*

NpHR, mouse genotype
TAG, ear tag number
ID, experiment identification number
SECTION, number of coronal brain section
AP, anteroposterior coordinate (in mm)
ML, mediolateral coordinate (in mm)
DV, dorsoventral coordinate (in mm)
TEST, behavioral test performed
NUCLEI, VP, ventral pallidum; SI, substantia innominata; HDB, hindbrain diagonal band; MCPO, magnocellular medial preoptic nucleus
-, undetermined fiber location (mouse lost implanted fibers before end of research protocol)
X, non-tested (mouse lost implanted fibers before end of research protocol).

A

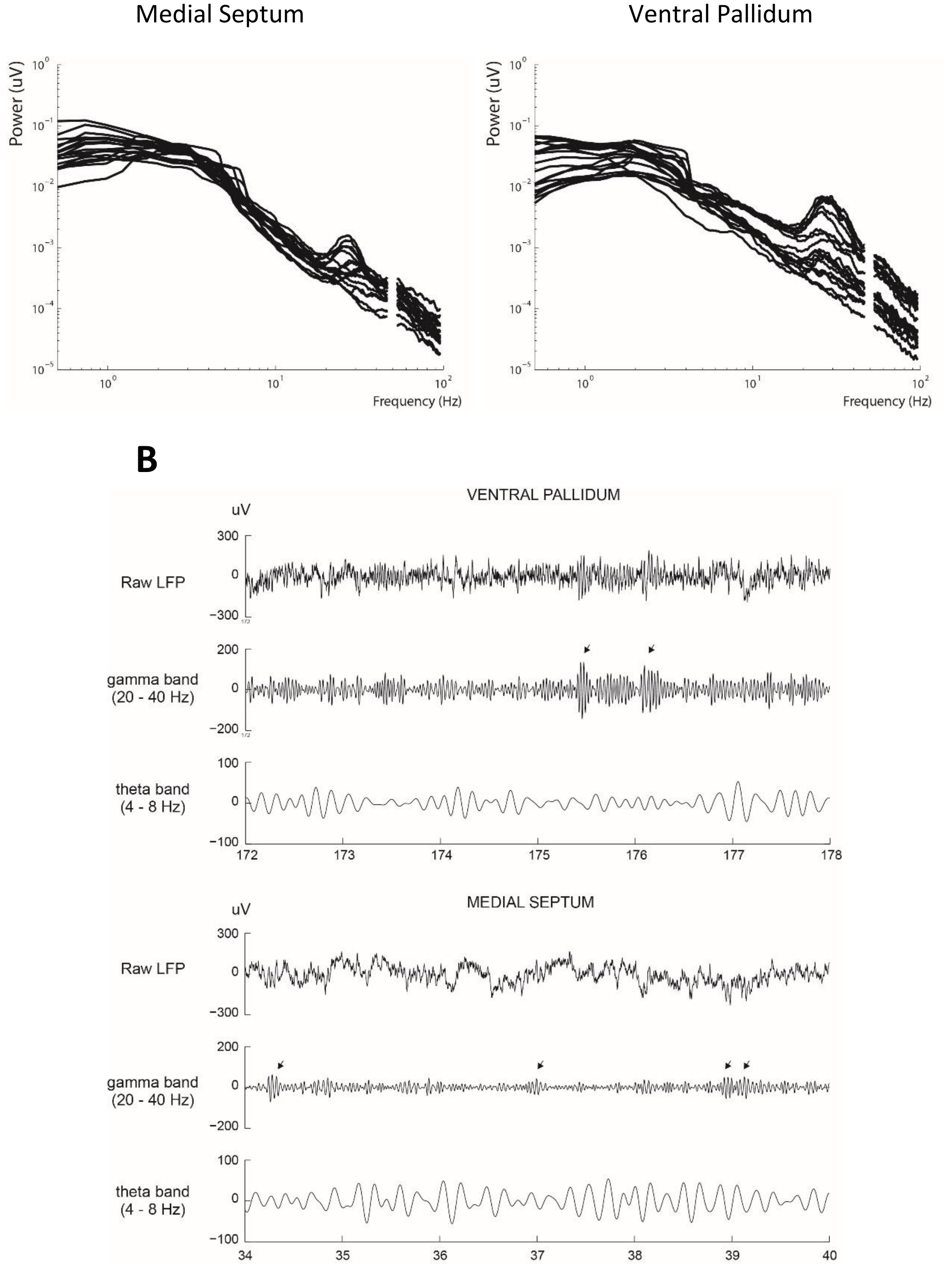

Supplementary Figure 1: **A,** LFP power spectral density for Medial Septum (7 animals, 18 sessions) and Ventral Pallidum (4 animals, 20 sessions). Note clear peaks in gamma band activity (around 30 Hz) but absence of prominent theta oscillations (4-8 Hz). 50 Hz artifact has been removed. **B,** Examples of LFP recorded in Ventral Pallidum (mouse NE58) and Medial Septum (mouse NE112) and filtered for gamma and theta bands. Arrows depict gamma events.

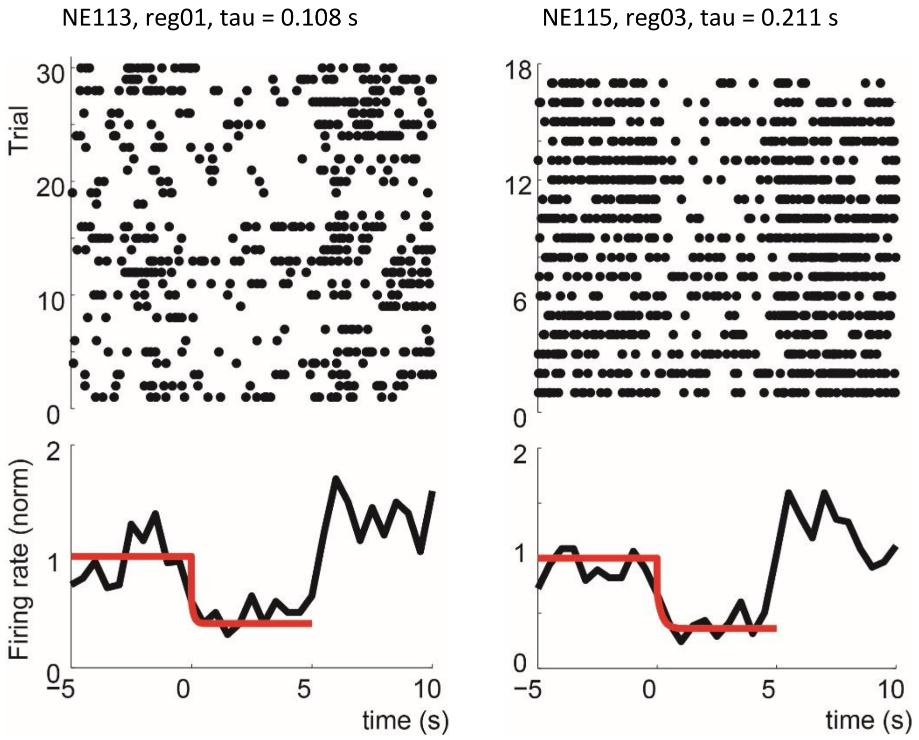

Supplementary Figure 2: Example putative somatostatin neurons recorded in the Medial Septum. Raster plots (top) and normalized peristimulus time histograms (bottom) for two neurons with fast (left) and slow (right) photoinhibition time constants (tau). Red line represents single exponential fitting. The variability of tau was large for inhibited cells, median= 0.33 s, IQR = 0.13 – 0.39 s. Data recorded from Ventral Pallidum and Medial Septum were pooled together because inhibition time constants were not different between populations (P = 0.7350, Wilcoxon rank sum test; VP, median: 0.28 s, IQR: 0.08 – 0.39 s; MS, median = 0.33 s, IQR: 0.19 – 0.39 s). In addition, we estimated inhibition latency by computing the decrease in firing rate (2 S.D. below the mean) within 25ms-bins after the onset of laser stimulation. We found that all putative somatostatin neurons detected by shuffling were inhibited within the first 25 ms of laser stimulation (median = 25 ms, QR = 0-50 ms) in about half of the trials (51.5%). Note that for trial-by-trial analysis not all sweeps could be considered as did not have enough spikes to compute a baseline preceding laser stimulation. Consistent results were found for 50-ms and 100-ms bins. Inhibition latency was not different between Ventral Pallidum and Medial Septum (P = 0.1913, Wilcoxon rank sum test). Such rapid, low-variability inhibition is consistent with the functional expression of NpHR in somatostatin cells.

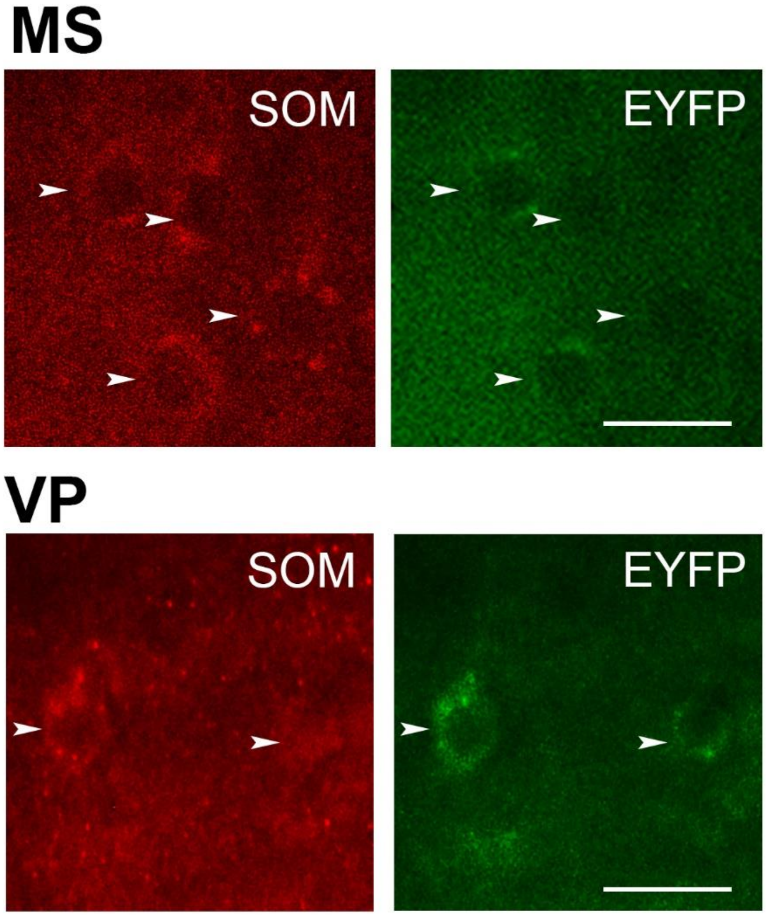

Supplementary Figure 3. Halorhodopsin expression in the basal forebrain. Fluorescent micrographs showing the expression of NpHR (EYFP) and somatostatin (SOM) in the Medial Septum and Ventral Pallidum of transgenic animals. Cell counting for the Medial Septum: EYFP+/SOM+, n = 226; EYFP+/SOM-, n = 52; EYFP-/SOM+, n = 58. Cell counting for the Ventral Pallidum was previously reported (Espinosa et al. 2017, Cereb Cortex). Scale bar 25 um.
